# RNA-Seq Based De Novo Transcriptome Assembly and Gene Discovery of *Cistanche deserticola* Fleshy Stem

**DOI:** 10.1371/journal.pone.0125722

**Published:** 2015-05-04

**Authors:** Yuli Li, Xiliang Wang, Tingting Chen, Fuwen Yao, Cuiping Li, Qingli Tang, Min Sun, Gaoyuan Sun, Songnian Hu, Jun Yu, Shuhui Song

**Affiliations:** 1 CAS Key Laboratory of Genome Sciences and Information, Beijing Institute of Genomics, Chinese Academy of Sciences, Beijing, China; 2 Core Genomic Facility, Beijing Institute of Genomics, Chinese Academy of Sciences, Beijing, China; 3 University of Chinese Academy of Sciences, Beijing, China; 4 HongKui CongRong Group, Alashan, Inner Mongolia, China; University of Western Sydney, AUSTRALIA

## Abstract

**Backgrounds:**

*Cistanche deserticola* is a completely non-photosynthetic parasitic plant with great medicinal value and mainly distributed in desert of Northwest China. Its dried fleshy stem is a crucial tonic in traditional Chinese medicine with roles of mainly improving male sexual function and strengthening immunity, but few mechanistic studies have been conducted partly due to the lack of genomic and transcriptomic resources.

**Results:**

In this study, we performed deep transcriptome sequencing in fleshy stem of *C*. *deserticola*, and about 80 million reads were generated using Illumina pair-end sequencing on HiSeq2000 platform. Using trinity assembler, we obtained 95,787 transcript sequences with transcript lengths ranging from 200bp to 15,698bp, having an average length of 950 bases and the N50 length of 1,519 bases. 63,957 transcripts were identified actively expressed with FPKM ≥ 0.5, in which 30,098 transcripts were annotated with gene descriptions or gene ontology terms by sequence similarity analyses against several public databases (Uniprot, NR and Nt at NCBI, and KEGG). Furthermore, we identified key enzyme genes involved in biosynthesis of lignin and phenylethanoid glycosides (PhGs) which are known to be the primary active ingredients. Four phenylalanine ammonia-lyase (PAL) genes, the first key enzyme in lignin and PhG biosynthesis, were identified based on sequences comparison and phylogenetic analysis. Two biosynthesis pathways of PhGs were also proposed for the first time.

**Conclusions:**

In all, we completed a global analysis of the *C*. *deserticola* fleshy stem transcriptome using RNA-seq technology. A collection of enzyme genes related to biosynthesis of lignin and phenylethanoid glysides were identified from the assembled and annotated transcripts, and the gene family of PAL was also predicted. The sequence data from this study will provide a valuable resource for conducting future phenylethanoid glysides biosynthesis researches and functional genomic studies in this important medicinal plant.

## Introduction

*C*. *deserticola* is a worldwide genus of perennial desert plants from the *Orobanchaceae* family, and is a completely non-photosynthetic species and usually grows underground holoparasitic plant [1]. It is parasitized on the roots of psammophyte *Haloxylon ammodendron* (*Chenopodiaceae*) [1, 2], which mainly inhabits deserts and semi-deserts due to its high tolerance to drought and salinity [1, 3]. *C*. *deserticola* shows strong resistance to harsh environmental conditions and is mainly distributed in Northwest China [4–6], especially in Inner Mongolia, Gansu and Xinjiang. It is considered to be an endangered wild species in recent years due to increased consumption by humans [5, 6]. *C*. *deserticola* which is often called desert ginseng is commonly known as desert-broomrape and the dried fleshy stem has been extensively used as a traditionally important tonic in China and Japan for many years [4, 7–10]. It was initially recorded in Shen Nong Ben Cao Jing (Dictionary of Chinese Materia Medica, 1977) [11] approximately 1800 years ago and was regarded as one of the main sources of the Chinese medicinal herba *Cistanche*.

The extracts of *C*. *deserticola* possess a wide range of medicinal functions, especially for use in improving sexual function, tonifying kidney, protecting liver, aperient activity, enhancing memory, immunomodulatory, antioxidative activity, anti-inflammatory, antiviral activity etc [7–10, 12–15]. The major bioactive components of *C*. *deserticola* are Phenylethanoid glycosides (PheGs, PhGs) [2, 9, 10, 14, 15]. To date, more than 20 phenylethanoid glycosides have been isolated from the succulent stem of *C*.*deserticola* [9, 14, 16]. Among them, acteoside and echinacoside are two main components with significant pharmacological activities [2, 16], and documented as the quality standards of *C*. *deserticola* in the Chinese pharmacopeia (2005 and 2010 editions). Three chemical components of PhGs are organic acid, saccharide and phenylethanoid, however, the details concerning phenylethanoid biosynthetic pathways remain poorly understood in *C*.*deserticola*.

Despite the commercial and medicinal importance of *C*.*deserticola*, the genomic and transcriptomic data of this species are very limited. There is no ESTs available in the NCBI database and the complete genome information for this species remains unavailable except for the chloroplast genome sequence [1]. The limited transcriptomic data hinder the study of PhG biosynthetic mechanisms. RNA-seq technology can generate sequences of the expressed parts of targeted genome [17] and identify genes [18] using the NGS technology platforms (such as Applied Biosystems SOLiD, Illumina HiSeq and Roche 454). It is becoming increasingly popular in transcriptome de novo assembly [19–22], since it is a cost-effective and powerful approach with high resolution and broad dynamic range [23–25], especially that it has an advantage to explore low abundance transcripts [26]. Because of the various advantages, RNA-seq is specifically attractive for non-model organisms with limited genetic resources [27–29]. But there is no any detailed research of *C*. *deserticola* transcriptome by RNA-seq.

In this study, we globally sequenced the stem transcriptome for *C*. *deserticola* using Illumina Hiseq2000 platform, and got 7.9G raw data. By assembly and annotation, we mined the genes involved in biosynthesis of PhG and the genes responsible for entire lignin biosynthesis. Our RNA-seq analysis generated the first *C*. *deserticola* consensus trancriptome and provided new insights into comprehensive understanding of the medicinal value of *C*. *deserticola*. Additionally, the method described here can be widely applied to profile transcriptomes to facilitate the discovery of genes involved in specific medicinal components biosynthesis pathway in other medicinal plant with very limited genomic resources.

## Materials and Methods

### Plant material collection

The fresh succulent stem for *C*. *deserticola* in excavation stage was collected from a plant base in BayanHot City of Alxa League in Inner Mongolia in northwestern China. The collecting permit was obtained from the owner (HongKui CongRong Group) of the plant base. The voucher specimen was deposited in the Core Genomic Facility at Beijing Institute of Genomics, Chinese Academy of Sciences. After cleaning, the succulent stem tissues were cut into small pieces and immediately frozen in liquid nitrogen, and then stored at -80°C until further processing.

### RNA extraction, cDNA library construction and Illumina sequencing

Total RNA was extracted from the succulent stem using TRIzol Reagent (Invitrogen Inc., California, USA) according to the manufacturer’s instruction. The resulting samples were treated with DNase I to remove any genomic DNA. Extracted RNAs were quantified using an Agilent 2100 bioanalyzer (Agilent Technologies) and checked for integrity using denaturing agarose gel electrophoresis with ethidium bromide staining. RNA samples with A260/A280 ratios between 1.9 and 2.1, RNA 28S:18S ratios higher than 1.0, and RNA integrity numbers (RINs) ≥ 8.5 were used in subsequent analyses.

The RNA-seq libraries were generated using Illumina Truseq RNA Sample Preparation Kits. Poly(A)+ RNA was isolated from total RNA using Dynal ligo(dT)25 beads according to the manufacturer’s instructions. Following purification, fragmentation buffer was added to break the mRNA into short fragments. First-strand cDNA was synthesized using these short fragments as templates, along with SuperScript III reverse transcriptase and N6 random hexamer primer. Second-strand cDNA was then synthesized using buffer, dNTPs, RNaseH and DNA polymerase I. The resulting double-stranded cDNA was subjected to end-repair using T4 DNA polymerase, DNA polymerase I Klenow fragment, and T4 polynucleotide kinase, and ligated to adapters using T4 DNA ligase. Adaptor-ligated fragments were purified using a QiaQuick PCR extraction kit and eluted with EB buffer. After analysis using agarose gel electrophoresis, suitable fragments were selected as templates for PCR amplification. Sequencing of the resulting cDNA library was carried out with an Illumina HiSeq 2000 system.

### Transcripts *de novo* assembly and gene expression quantification

Raw reads generated from sequencing was cleaned by removing the adaptor sequences (ATCTCGTATGCCGTC) using in-house method. We then carried out a stringent low-quality filtering process. Firstly, bases with phred quality score lower than 20 would be trimmed from the 3’end of the sequence, until running into one base with a higher quality (≥20). If reads length was short than 50bp, it would be discarded. Secondly, reads will be further filtered by the criterion that 70% bases in one read have high quality scores (≥20). Thirdly, only paired-end reads were used for further assembly. *De novo* transcript assembly was conducted using Trinity release_20130216 [30] which consisted of three successive software modules: Inchworm, Chrysalis, and Butterfly. The assembly parameters were set as below:—seqType fq—JM 300G —min_contig_length 200—CPU 20—inchworm_cpu 20—bflyCPU 20.

To quantify transcript abundance, the sequenced pair-end reads were re-aligned to the assembled transcripts using a script in Trinity [31]. Mapped reads were used for quantification by RSEM (RNA-Seq by Expectation Maximization) software [32]. Gene or isoform abundance was represented by the fragment per kilobase of transcript per million fragment mapped (FPKM) value, those transcripts with FPKM value equal or larger than 0.05 were defined as expressed.

### Functional annotation of expressed transcripts

For there is no any gene annotation sets of *C*. *deserticola* except for chloroplast genome [1]. We annotated the expressed transcripts by comparing to Genbank Nt, Genbank Nr, and TAIR10_pep_20101214_updated datasets separately using BLAST program (E< = 1e^-20^). Meanwhile, all expressed transcripts were translated into potential proteins according to ORF prediction by TransDecoder [30] and predicated for the conserved domains based on Pfam database [33].

### Gene Ontology and KEGG pathway annotation

By sequence similarity alignment to Uniprot database (http://www.uniprot.org/), the Gene Ontology (GO) annotation of all assembled transcripts was obtained by using association file downloaded from (ftp://ftp.ebi.ac.uk/pub/databases/GO/goa/UNIPROT/gene_association.goa_uniprot.gz). GO terms clustering of expressed genes were conducted by using custom scripts, and we annotated genes at the fourth level for the CC, BP and MF categories separately.

KEGG pathway information was assigned for all predicted proteins sequences using online tool KAAS (KEGG Automatic Annotation Sever) [34]. Sequences in fasta format were submitted to KAAS request, and the resulting files of all pathways information related to *C*. *deseticola* stem transcriptome were downloaded. 13 plant organisms’ gene data sets in KEGG were used for annotation using the BBH (bi-directional best hit) method.

### RT-qPCR analysis

After digestion with DNase I, approximately 5μg of total RNA was converted into first-strand cDNA via the reverse-transcription reaction with oligo(dT)_15_ primers and GoScript Reverse Transcription System (Promega). The cDNA products were then diluted 10-fold with nuclease free deionized water before use as a template in real-time PCR. Specific cDNAs were amplified by GoTaq 2-Step RT-qPCR system (Promega) in a volume of 20 ul. PCR amplification was performed at the annealing temperature of 60°C with the 7500 Real-Time PCR Detection System (Applied Biosystems) according to the manufacturer’s instructions. Relative transcript abundances were calculated by the comparative cycle threshold method with gene “comp10579_c0” as an internal standard, using the 7500 Manager software.

Primer pairs for RT-PCR were designed based on online software (http://primer3.ut.ee/) and are listed in S1 Dataset.

## Results

### RNA sequencing and *de novo* transcriptome assembly of *C*. *deserticola* fleshy stem

**Fig 1:**
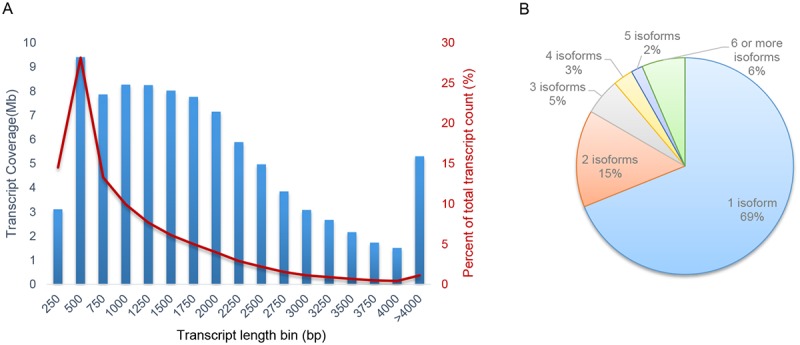
Distribution of assembled transcript length and statistic of isoform pattern. (a) Distribution of assembled transcript length binned at 250bp intervals. (b) The percentage of transcript isoform pattern ranging from a single isoform to 6 or more isoforms in a single assembled gene.

Stem of *C*. *deserticola* has been extensively used as a traditionally important tonic in China and Japan for many years. To obtain a global overview of gene expression in the *C*. *deserticola* fleshy stem, we collected *C*. *deserticola* stem samples of the same plant base in 2013 and 2014, respectively. Total RNAs were extracted and polyA+ RNAs were purified for constructing paired-end RNA-seq libraries. 79,433,734 and 86,019,176 pair-end reads corresponding to nearly 8 billion and 8.6 billion bases of sequence were obtained using Illumina HiSeq 2000 sequencing platform in 2013-year and 2014-year samples (Table 1). After removing adaptor sequences and filtering low quality reads (see details in Methods), 64,831,040 high quality pair-end reads in 2013-year sample were used for *de novo* transcriptome assembly. Using Trinity sequence assembler [30], 51,719 genes and 95,787 transcript sequences were generated with transcript lengths ranging from 200 bp to 15,698 bp. The average length of assembled transcripts is 950 bases and the N50 length is 1,519 bases. The number of transcript in different lengths revealed that 57.32% of the assemble transcript were about 500-bp or longer (Fig 1A). High quality pair-end reads in 2014-year sample were mapped to the assembled transcriptome. Besides, we found that the transcript number for each assembled genes varied and 69% genes with one expressed isoform while 31% genes expressed two or more transcripts (Fig 1B).

**Table 1:** Description of sequencing data and de novo assembly.

	2013-year sample	2014-year sample
**Read processing**		
Raw reads# (2×101 bp)	79,433,734	86,019,176
De_adaptored reads#	73,294,304	83,580,765
High quality reads#	69,482,486	80,071,469
Paired reads#	64,831,040	75,350,594
Bases for assembly (Gb)	6.54	-
**Assembly**		
Assembled reads#	53,212,524	
Assembled transcripts#	95,787	
Assembled components#	51,719	
Assembled bases#	90,989,781	
Transcript N50 (bp)	1,519	
**Mapping**		
Mapped reads#	50,588,722	53,988,016
Uniquely mapped reads#	34,206,716	32,488,394
**Expression** ^**a**^		
Expressed transcripts#	63,957	52,857
Expressed transcripts with protein-encoding potentials#	30,098	23,433

^a^Transcripts with FPKM value equal or larger than 0.5 are defined as expressed.

### Expression quantification and functional annotation of assembled transcripts

Gene or transcript abundance was quantified using RSEM package, in which the sequenced reads were re-aligned to the assembled genes or transcripts sequences using Bowtie and those mapped reads were used for quantification. FPKM value for each gene or transcript was calculated, and finally we identified 63,957 and 52,857 actively expressed transcripts (FPKM value ≥ 0.5) in *C*. *deserticola* fleshy stem samples in 2013 and 2014, respectively. 44,776 transcripts (70.01% in 2013-year sample, 84.71% in 2014-year sample) were commonly expressed in the two replicates, and the correlation (Pearson correlation coefficient: 0.91979) of their expression data was showed in S1 Fig. The sequencing raw data had been uploaded to NCBI SRA database (accession numbers: SRX857402 and SRX858938). We used expressed genes identified in 2013-year sample for further analysis. Functional annotation information for all expressed transcripts was obtained using two methods. Firstly, all expressed transcripts were aligned to known nucleotide (GenBank nt) and peptide sequence databases (GenBank nr and Arabidopsis peptide) separately by BLAST algorithm. Out of 63,957 expressed transcripts, 29,220 (45.7%) were annotated and showed homology to sequences in any of the three subject databases with *E*-value cutoff 1e-20. Meanwhile, the candidate coding regions for all expressed transcript sequences were predicted using TransDecoder software and the longest ORFs for each transcript were used for Pfam domain search. As a result, 21,358 (33.4%) transcripts were annotated based on Pfam database. Overall, 30,098 (47.1%) transcripts were significantly matched to known genes in the public databases by combining the two methods above. The complete expressed transcripts list with function annotation were showed in supplemental data (S2 Dataset).

We surveyed the top 20 most highly expressed transcripts (Table 2) corresponding to 18.99% of all sequencing reads, and found that most of them are genes responding to abiotic stress stimulus. Dehydrin (DHNs), a class of hydrophilic and thermostable stress proteins with a high number of charged amino acids that belong to the Group II Late Embryogenesis Abundant (LEA) family, is the most highly expressed genes. Three different Dehyrin transcripts (comp28713_c0_seq1/2/4) were detected highly expressed in fleshy stem which may be involved in protecting cells from damage caused by drought stress. Other stress-related genes such as heat shock protein, pathogen-related protein and metallothionein were also found expressed highly, which may be related to its severe survival environment. Additionally, some constitutive genes including 26S ribosomal RNA gene (comp22329_c2_seq1), auxin-repressed/dormancy-associated protein (comp20999_c0_seq1), ADP-ribosylation factor (comp20499_c0_seq1) were also highly transcribed.

**Table 2:** The top 20 highly expressed genes in fleshy stem tissue of *C*. *deserticola*.

Transcript id	FPKM	Predicted Function
comp28713_c0_seq2	17733.25	Dehydrin
comp22282_c0_seq1	17066.35	Avicennia marina class I type 2 metallothionein
comp22329_c2_seq1	16735.83	Valeriana officinalis 26S ribosomal RNA gene
comp29412_c2_seq2	14550.58	Oryza sativa Japonica Group clone KCG115D09 17.4 kDa heat shock protein mRNA
comp25013_c0_seq1	13532.52	pathogen-related protein STH-2 [Salvia miltiorrhiza]
comp29412_c1_seq4	12074.82	*^a^
comp24506_c0_seq9	11779.81	*
comp29412_c1_seq3	7156.45	*
comp10462_c0_seq1	6667.79	*
comp20999_c0_seq1	6596.5	Citrus cv. Shiranuhi putative auxin-repressed/dormancy-associated protein mRNA
comp10453_c0_seq1	6045.73	MLP-like protein 28-like; Pathogenesis-related protein Bet v I family
comp28713_c0_seq4	5815.95	Dehydrin
comp29412_c1_seq1	4007.95	*
comp20237_c0_seq6	3723.08	Coffea canephora dehydrin (DH3) mRNA, complete cds
comp26495_c2_seq5	3460.12	Carthamus tinctorius ubiquitin (UBQ) mRNA
comp28713_c0_seq1	3381.59	Dehydrin
comp22515_c0_seq1	3313.78	Sesamum indicum polyphenol oxidase (PPO) mRNA
comp14988_c0_seq1	2811.77	Extensin-like region
comp20499_c0_seq1	2748.74	Salvia miltiorrhiza ADP-ribosylation factor mRNA
comp30181_c0_seq1	2710.42	*

^a^The signal of “*” shows that the transcript was not annotated by public databases.

### Functional classification of all expressed transcripts based on Gene Ontology and KEGG databases

Gene Ontology (GO) annotation was obtained from Uniprot annotation and identities association file. In total, 20,907 transcripts, accounting for 32.69% of the total expressed sequences, were assigned to 1,745 functional terms. Of the total functional GO terms, assignments to the biological process made up the majority (1,116, 63.95%) followed by cellular component (329, 18.85%) and molecular function (300, 17.20%). The assigned functions of expressed transcripts covered a broad range of GO categories, and the top 10 GO terms with most annotated transcripts were listed in Table 3. And we provide all expressed transcripts distribution in three Gene Ontology categories (Molecular Function, Cellular component and biological process) in supplemental file (S3 Dataset). GO terms related to binding functions and transferase activity were predominantly represented in the molecular function category. About the binding functions, cation binding (4,394 transcripts) represented the most abundant, followed by nucleotide/nucleoside binding (3,404 transcripts in average) and protein binding (2,422 transcripts). While in transferase activity group, the most is those with transferring phosphorus-containing groups (2,256 transcripts, 65.77%). Among the cellular component category, transcripts were more located to intracellular (10,581 transcripts in average), while among the biological process category, transcripts were more involved in biopolymer metabolic process (6,683 transcripts in average), followed by regulation of cellular process (4,841 transcripts), gene expression (4,678 transcripts) and transport (3,512 transcripts).

**Table 3:** The top 10 GO terms with the highest transcript number in each of the three ontologies.

Functional terms	GO Accession Number	Transcripts Count
**Molecular Function**		
cation binding	GO:0043169	4,394
purine nucleotide binding	GO:0017076	4,105
nucleotide binding	GO:0000166	4,065
ribonucleotide binding	GO:0032553	3,915
purine nucleoside binding	GO:0001883	3,680
transferase activity	GO:0016740	3,430
hydrolase activity	GO:0016787	3,052
protein binding	GO:0005515	2,422
transferase activity, transferring phosphorus-containing groups	GO:0016772	2,256
DNA binding	GO:0003677	2,233
**Cellular component**		
intracellular	GO:0005622	13,541
intracellular part	GO:0044424	13,471
intracellular organelle	GO:0043229	11,766
intracellular membrane-bounded organelle	GO:0043231	11,083
cytoplasm	GO:0005737	10,020
cytoplasmic part	GO:0044444	8,271
membrane	GO:0016020	6,738
intracellular organelle part	GO:0044446	5,915
membrane part	GO:0044425	5,079
intrinsic to membrane	GO:0031224	4,073
**Biological process**		
biopolymer metabolic process	GO:0043283	9,927
cellular macromolecule metabolic process	GO:0044260	9,371
cellular biosynthetic process	GO:0044249	7,043
nucleobase, nucleoside, nucleotide and nucleic acid metabolic process	GO:0006139	6,380
protein metabolic process	GO:0019538	5,703
macromolecule biosynthetic process	GO:0009059	5,031
regulation of cellular process	GO:0050794	4,841
gene expression	GO:0010467	4,678
transport	GO:0006810	3,512
regulation of metabolic process	GO:0019222	3,325

**Fig 2:**
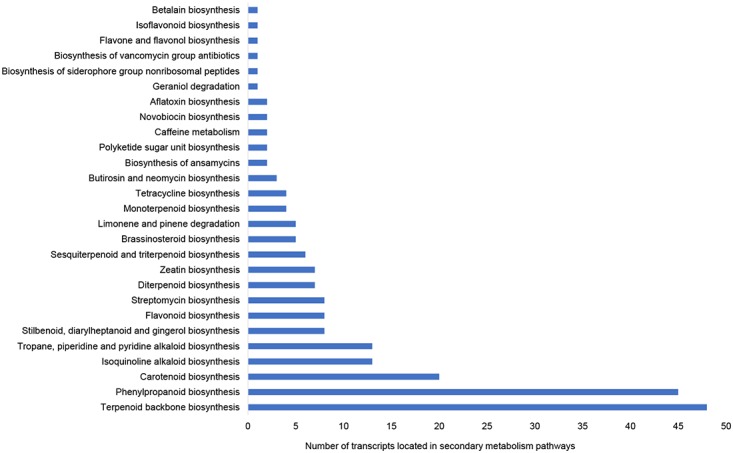
The KEGG pathways involved in secondary metabolism in *C*. *deserticola*. KEGG classification based on secondary metabolite categories sorted by counts of transcripts in a single pathway.

In order to mine genes involved in biosynthesis of lignin and PhG, 21,358 non-redundant potential protein sequences were searched against gene sequences of 13 plant organisms in KEGG database, and they were assigned to 275 KEGG pathways with at least 5 hits. The top 10 pathways with most aligned sequences were listed in Table 4. Most pathways were involved in primary metabolic processes, such as amino acid or protein metabolism (ko01230, ko04141 and ko04120), carbohydrate metabolism (ko01200 and ko00500) and nucleotide or nucleoside metabolism (ko03018, ko00230, and ko00240). Besides, there are 27 secondary metabolism related pathways (Fig 2), such as terpenoid backbone biosynthesis, phenylpropanoid biosynthesis, carotenoid biosynthesis, isoquinoline alkaloid biosynthesis and tropane, piperidine and pyridine alkaloid biosynthesis. These results provide further indication that active metabolic processes were underway in the *C*. *deserticola* stem tissue. All expressed transcripts associated with KEGG pathways were listed in supplemental file (S4 Dataset). Although there are some significantly changed pathways between *C*. *deserticola* and other plants, such as rice [35] (S5 Dataset), our main goal in this study aims to reveal the whole transcriptome profile of *C*. *deserticola* stem, and to picture the related pathways of PhGs biosynthesis which could be useful for guiding the cultivation.

**Table 4:** The top 10 KEGG pathways with most protein sequences.

KO NO.	Protein count	KEGG pathway name
ko01230	164	Biosynthesis of amino acids
ko01200	161	Carbon metabolism
ko00230	116	Purine metabolism
ko04141	108	Protein processing in endoplasmic reticulum
ko03013	100	RNA transport
ko04120	97	Ubiquitin mediated proteolysis
ko00500	90	Starch and sucrose metabolism
ko00240	84	Pyrimidine metabolism
ko03018	80	RNA degradation
ko05169	79	Epstein-Barr virus infection

### Candidate genes encoding enzymes involved in the biosynthesis of lignin

**Fig 3:**
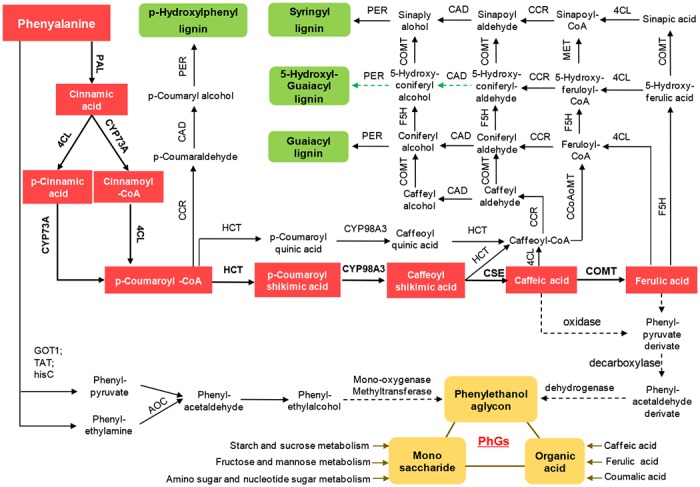
Detailed pathways and genes involved in the synthesis of lignin and PhGs in *C*. *deserticola* stem. The top and bottom panels showed the detailed pathways and genes involved in the biosynthesis of four types lignin and PhGs, respectively. Red diamonds represent the common pathway shared by lignin and PhGs. Green dotted lines were used to represent the synthesis pathway of 5-Hydroxyl-Guaiacyl lignin, which was identified in COMT deficient plants. **Abbreviation:** PAL(Phenylalanine ammonialyas), 4CL(4-coumarate-CoA ligase), CYP73A(trans-cinnamate 4-monooxygenase), CCR(cinnamoyl-CoA reductase), HCT(shikimate o-hydroxycinnamoyltransferase), F5H(ferulate-5-hydroxylase), COMT(3-O-methyltransferase), CCoAOMT(caffeoyl-CoA O-methyltransferase), CAD(cinnamyl-alcohol dehydrogenase), PER(peroxidase), CYP98A3(coumaroylquinate/coumaroylshikimate 3'-monooxygenase), CSE(caffeoylshikimate esterase), GOT1(aspartate aminotransferase), TAT(tyrosine aminotransferase), hisC(histidinol-phosphate aminotransferase) and AOC3(primary-amine oxidase).

Lignin is the second most abundant natural terrestrial polymer in plant kingdom, composing up to one-third of the material found in plant cell walls [36, 37]. As an important component of cell wall, lignins help water transport, provide mechanical support and structural integrity, and defend against pathogens and herbivores [38–40]. Those roles of lignin are much valuable to support underground erective growth of *C*. *deserticola* in desert. In this study, we presented the complete picture of lignin biosynthesis pathways in *C*. *deserticola* (Fig 3), in which the lignin monomers are biosynthesized from phenylalanine through a series of enzymatic reactions, including hydroxylation, methylation, reduction, and oxidative polymerization process. Lignin biosynthesis-related enzymes were detected for three mainly synthesized forms in vascular tissue (p-hydroxyl-phenyl (H), guaiacyl (G) and syringyl (S) lignin) and 5-hydroxyl-guaiacyl lignin which was only identified in COMT (caffeic acid 3-O-methyltransferase, EC 2.1.1.68) deficient (such as knock-down) plants [41, 42].

Phenylalanine ammonialyas (PAL, EC 4.3.1.24) is the first key enzyme in lignin biosynthesis pathway (Fig 3) which transforms phenylalanine into cinnamic acid by non-oxidative deamination [43, 44]. A total of 6,297 PALs reads were sequenced and 7 PAL transcripts were assembled in *C*. *deserticola* (Table 5). By sequence similarities comparison, we found that 4 of them (comp28550_c1_seq1/2/3/5) had more than 95% similarity with the known mRNA sequence of *C*. *deserticola* (gi|289595227|gb|ADD12041.1|), while comp28550_c1_seq4 and comp25940_c0_seq1 had 77% and 82% similarities, respectively. ORF prediction revealed 5 transcripts had potentials of encoding proteins and carried with aromatic amino acid lyase domain (PF00221.14). Among them, only the comp28550_c1_seq4 transcript could encode a complete protein sequence of 718 amino acid residues. It has been reported that PAL was encoded by a small multigene family in most plant species, such as 4 in Arabidopsis thaliana [39, 45], 5 in Populus trichocarpa [46, 47], 3 in Scutellaria baicalensis [48], and 7 Cucumis sativus [43, 49] etc. Our phylogenetic analysis suggested that there were 4 PAL encoding genes in *C*. *deserticola* and we named them CdPAL1, CdPAL2, CdPAL3 and CdPAL4, respectively (S2 Fig). 4-coumarate-CoA ligase (4CL, EC 6.2.1.12) and trans-cinnamate 4-monooxygenase (CYP73A, EC 1.14.13.11) are two enzymes responsible for transforming cinnamic acid to p-coumaroyl-CoA in two reverse orders. They are also in backbones, and their expression FPKM values are 39.57 and 51.93, respectively.

**Table 5:** Phenylalanine ammonia lyase (PAL) mRNA in de novo assembly transcriptome of *C*. *deserticola*.

Transcript ID	Length	Matched length to known sequence ^a^	Identity to known sequence ^a^ (%)	Supported Read#	Length of predicted protein	Protein type	Range	Lyase aromatic Domain
comp28550_c1_seq1	447	239	95.4	354	*^b^	*	*	N
comp28550_c1_seq2	2076	1868	98.02	1970	606 aa	5prime_partial	1–1818	Y
comp28550_c1_seq3	2077	1868	98.02	1971	606 aa	5prime_partial	1–1818	Y
comp28550_c1_seq4	2506	1729	82.73	2726	718 aa	complete	286–2439	Y
comp28550_c1_seq5	448	239	95.4	355	*	*	*	N
comp25940_c0_seq1	1499	1021	77.52		460 aa	3prime_partial	1–1380	Y
comp25940_c1_seq1	820	-	-		240 aa	5prime_partial	2–721	Y

^a^All transcript sequences were compared with the *C*. *deserticola* known complete CDS sequence (gi|289595227|gb|ADD12041.1|) of phenylalanine ammonia lyase (PAL). The sequence of ‘comp25940_c1_seq1’ had little similarity with the *C*. *deserticola*, but with 78.24% similarity with *Camellia chekiangoleosa* PAL sequence.

^b^The signal of “*” represents no results produced.

**Fig 4:**
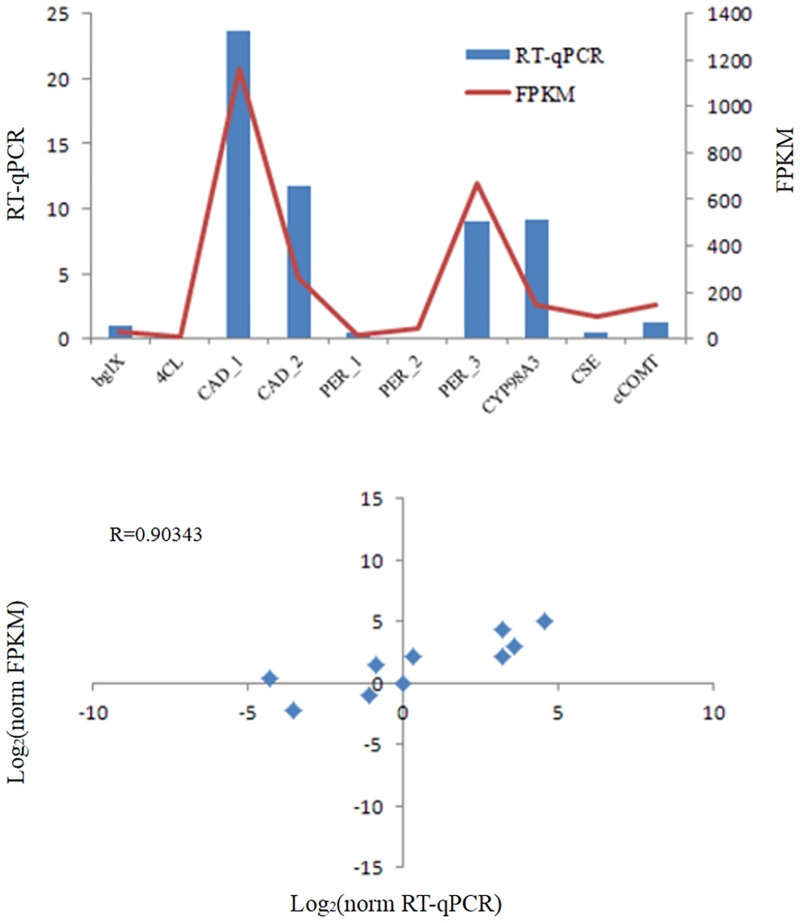
RT-qPCR analysis. Real-time PCR results for 10 selected genes related to lignin biosynthesis pathway. Plot in the top panel of the figure presents RNA-seq (line graph) and qPCR (bar graph) quantification results, and the bottom panel shows their high correlations (r>0.9). Corresponding gene names and unique gene identifier numbers in the assembly are bglX (comp10579_c0), 4CL (comp26895_c0), CAD_1 (comp30193_c0), CAD_2 (comp30398_c0), PER_1 (comp21305_c0), PER_2 (comp25087_c0), PER_3 (comp30272_c0), CYP98A3 (comp26902_c8), CSE (comp19323_c0), and cCOMT (comp28020_c1). (Notes: as comp30193_c0 and comp30398_c0 were both annotated as CAD gene, we added subscripts to differentiate them. The same thing was done to comp21305_c0, comp25087_c0 and comp30272_c0, which were all annotated as PER gene).

The four type lignins were biosynthesized by different pathways which were controlled by three key enzymes, cinnamoyl-CoA reductase (CCR, EC 1.2.1.44), shikimate o-hydroxycinnamoyltransferase (HCT, EC 2.3.1.133), and ferulate-5-hydroxylase (F5H, EC 1.14.-.-). CCR was reported as a control point of lignins pathway [50, 51] which catalyzed X-CoA (X including p-coumaroyl, caffeoyl, feruloyl, 5-hydroxyl-feruloyl and sinapoyl) into Y-aldehyde (Y including p-coumar, caffeyl, coniferyl, 5-hydroxyl-coniferyl and sinap), while HCT catalyzed p-coumaroyl-CoA to p-coumaroyl shikimic acid/p-coumaroyl quinic acid. The two enzymes, just like a switch, regulated biosynthesis of P-hydroxyl-phenyl lignins or the other three type lignins. F5H was another branch switch that regulated syringyl lignin and 5-hydroxyl-guaiacyl lignin. Other important enzymes including caffeic acid 3-O-methyltransferase (COMT, EC 2.1.1.68), caffeoyl-CoA O-methyltransferase (CCoAOMT, EC 2.1.1.104) and cinnamyl-alcohol dehydrogenase (CAD, EC 1.1.1.195) were also detected expressed. Detailed expression information was listed in Table 6. These enzyme genes identified in this study will provide a valuable resource for functional genomic studies in this important medicinal plant. 10 genes related to lignins biosynthesis pathway in Table 6 were selected for RT-qPCR verification to confirm our RNA-seq results (Fig 4), and their high correlations (Pearson correlation coefficient: 0.90343) indicated high accuracy and reproducibility of our transcriptome analysis. S1 Dataset lists the primer sequences used in this analysis.

**Table 6:** Expression profile of related genes in biosynthesis of lignin and PhGs.

Description	EC Number	ORF ID	FPKM
phenylalanine ammonia-lyase	4.3.1.24	comp25940_c0	3.93
		comp28550_c1	22.54
beta-glucosidase	3.2.1.21	comp15401_c0	19.27
		comp27218_c0	1.65
		comp10579_c0	34.08
		comp28293_c0	7.76
		comp56_c0	0.92
4CL;4-coumarate—CoA ligase	6.2.1.12	comp26895_c0	7.2
		comp29240_c0	32.37
CCR;cinnamoyl-CoA reductase	1.2.1.44	comp26393_c2	3.89
CYP73A;trans-cinnamate 4-monooxygenase	1.14.13.11	comp10963_c0	51.93
cinnamyl-alcohol dehydrogenase	1.1.1.195	comp20951_c0	31.67
		comp27272_c0	70.95
		comp27835_c0	18.94
		comp29267_c0	1.77
		comp30193_c0	1159.57
		comp30398_c0	259.8
peroxidase	1.11.1.7	comp10543_c0	42.74
		comp10650_c0	41.53
		comp119949_c0	1.7
		comp13993_c0	0.8
		comp21153_c0	1.94
		comp21305_c0	17.16
		comp25087_c0	45.26
		comp28355_c2	43.19
		comp28694_c0	9.8
		comp29269_c3	3.47
		comp29883_c2	13.01
		comp30017_c0	0.78
		comp30161_c0	6.59
		comp30272_c0	665.8
		comp7217_c0	0.71
HCT; shikimate O-hydroxycinnamoyltransferase	2.3.1.133	comp73580_c0	3.4
CYP98A3, C3'H; coumaroylquinate (coumaroylshikimate) 3'-monooxygenase	1.14.13.36	comp22365_c1	10.05
		comp23009_c0	1.46
		comp26902_c8	150.22
CSE; caffeoylshikimate esterase	3.1.1.-	comp19323_c0	96.8
COMT; caffeic acid 3-O-methyltransferase	2.1.1.68	comp29821_c1	39.63
caffeoyl-CoA O-methyltransferase	2.1.1.104	comp28020_c1	145.37
CYP84A, F5H;ferulate-5-hydroxylase	1.14.-.-	comp52365_c0	3.83
GOT1;aspartate aminotransferase	2.6.1.1	comp14836_c0	54.81
		comp18427_c0	3.21
		comp28117_c0	13.99
		comp29202_c0	10.29
TAT; tyrosine aminotransferase	2.6.1.5	comp29792_c0	6.33
hisC; histidinol-phosphate aminotransferase	2.6.1.9	comp18748_c0	21.59
AOC3, AOC2, tynA; primary-amine oxidase	1.4.3.21	comp18613_c0	68.63
		comp29232_c0	100

### Candidate genes encoding enzymes involved in the biosynthesis of PhGs

Phenylethanoid glycosides (PhGs) are known to be the primary active ingredients in *C*. *deserticola* with activities of improving sexual potency, scavenging free radical and anti-aging [7–10]. Three chemical components of PhGs are organic acid, saccharide and phenylethanol aglycon (Fig 3). The organic acid including caffeic acid, ferulic acid and coumalic acid are products of phenylpropanoid biosynthesis pathway. The components of saccharide including glucose and rhamnose are products of carbohydrate metabolism pathways, such as starch and sucrose metabolism, amino sugar and nucleotide sugar metabolism, fructose and mannose metabolism etc. However, the biosynthesis pathway of phenylethanol part is not clear yet. Here, we proposed two possible phenylethanol biosynthesis pathways based on our sequence data. One is the reported caffeic acid or ferulic acid pathway, also known as cinnamiv acid pathway which is similar to the lignin biosynthesis backbone pathway. Another is based on phenylalanine metabolism pathway (Fig 3), in which the phenylalanine to phenylethanol was achieved by a known ‘Enrlich pathway’ that was first found in yeast one century ago and validated in petunia flowers [52, 53], tomato [54], and rose [55, 56]. Four enzyme genes encoding aspartate/tyrosine aminotransferase, histidinol-phosphate aminotransferase, and primary-amine oxidase which are responsible for the conversion of phenylalanine to phenylethanol were detected expressed in stem of *C*. *deserticola*. The product of phenylethanol may be further oxidized by monooxygenase or methylated by methytransferase into its derivates (phenylethanol aglycon) which took part in PhG biosynthesis. In summary, two putative biosynthesis pathways of phenylethanol aglycon were proposed for *C*. *deserticola* but still needs more study in further.

## Discussions

Recent years, plant genomics have developed rapidly with the application of next-generation sequencing technology, while few researches have been focused on the genomics of desert medicinal plants [57]. It is urgently necessary to perform genomic or transcriptomic research to understand its adaption to drought and salinity environment and the biosynthesis pathway of the major bioactive components. The *de novo* transcriptome discovery for some medical plants, such as *Panax ginseng* [58], *Ginkgo biloba* [59] and *Glycyrrhiza uralensis* [60] have been first exploited using Roche 454 platform for its long read length. Because of the effectively assembly ability with short reads, especially the advantaged paired-end reads [23, 61], Illumina-based transcriptome sequencing and assembly has also been extensively used for model [20, 22, 62–64] and non-model organisms [17, 19, 21, 65, 66]. In the present study, we generated about 8G of 101 bp paired-end reads and produced longer unigene sequences with 725 bp average length. Large-scale stem-specific transcriptome data could provide useful reference data and be used to mine secondary metabolism of bioactive components of *C*. *deserticola*. There is 81.62% of the total raw reads passed stringent quality filters (including adaptor trimming and low-quality reads discarding) before assembly, suggesting the high-quality of our sequencing data, and in which 82.08% of the high-quality reads were useful for assemble. Other reads failed to use for assemble may come from sequencing error [67], assembly parameters [23] et al. Those unused high-quality reads remained helpful to improve *de novo* assembly combined with longer reads from other platform (such as Roche 454) in the future.

A great number of assembled transcripts (30,098) showed high sequences similarities to know genes in public databases, suggesting that our Illumina-based paired-end data covered a substantial fraction of transcripts of *C*. *deserticola*. Transcripts with no BLAST hits may due to 3’ or 5’ untranslated regions, non-coding RNA [23] or new gene sequences of *C*. *deserticola*. The expressed transcripts were annotated to a wide range of GO categories and KEGG pathways (Tables 3 and 4), in which many transcripts were assigned to secondary metabolism related pathways. As we known, phenylpropanoid can function as inducible antimicrobial compounds with great salutary for an underground lifestyle [1], and also act as signal molecule in plant-microbe interactions besides for its medicinal utility [68, 69]. Terpenoid is used for biosynthesis of bioactive components (such as 6- deoxycatalpol) [70]. We found genes involved in the phenylpropanoid and terpenoid backbone biosynthesis pathway were highly abundant in *C*. *deserticola*. More importantly, the discovery of well represented pathways of lignin biosynthesis (Fig 3) indicated the active metabolic process of lignin in *C*. *deserticola* stem. All known enzyme genes involved in biosynthesis of lignin (Fig 3) were detected expressed, and four key enzymes including PAL, CCR, HCT and F5H had lower expression abundance (FPKM 26.47, 3.89, 3.4 and 3.83, respectively) compared with other enzyme genes (Table 6). Whether or not the expression change of those three genes could influence lignin production in *C*. *deserticola* is worthy of further study. PAL is a key enzyme in lignin biosynthesis and also involving in the biosynthesis of phenylpropanoid, resveratrol, flavonoid and coumarin [71–74]. We detected four distinct PAL genes in *C*. *deserticola* genome (S2 Fig) which was coincident with that PAL was encoded by a small multigene family [39, 43, 45–49] and further proved it may play important roles in metabolic carbon flux.

PhG is the primary active ingredients in *C*. *deserticola*. Genes involved in the biosynthesis of phenylethanol are important for the quality of *C*. *deserticola*. We deduced two different biosynthesis pathways of phenylethanol and 17 enzyme genes involved in PhG biosynthesis in *C*. *deserticola* stem. The possible post-caffeic/ferulic acid processes (Fig 3) were also deduced for the first time based on structural formula of intermediates and catalytic properties of corresponding enzymes, in which the caffeic/ferulic acid would be first oxidized into phenyl-pyruvate derivate; then, the carboxyl group was deprived by decarboxylases; finally, aldehyde group were converted back into alcohol group by dehydrogenase. This is the first application of Illumina paired-end sequencing technology to investigate the whole transcriptome of *C*. *deserticola* and to assemble RNA-seq reads without a reference genome. This study will provide useful resources and gene sequences for functional genomics and proteomics research on *C*. *deserticola* in future.

## Conclusions

In this study, we profiled the transcriptome of *C*. *deserticola* stem based on high-throughput sequencing data, and identified genes involved in biosynthesis pathways of lignin and also inferred potential biosynthesis pathway of PhGs for the first time, which will certainly accelerate the understanding of the ambiguous physiological processes and the great medicinal value in molecular level. Up to now, this is the first attempt to *de novo* assemble the whole transcriptome of *C*. *deserticola* stem and to detect biosynthesis pathway of medicinal components using Illumina-based sequencing datasets. Our study may promote the development of natural medicines and the selection of cultivars with medicinal traits.

## Supporting Information

S1 Fig Correlation between RNA-seq expression data of two replicated *C*. *deserticola* samples. Normalized read count of two replicates (2013-year sample and 2014-year sample) were plotted, and high correlation showed reproducibility of our RNA-seq data.
(TIF)

S2 Fig Phylogenetic analyses of PAL genes in *C*. *deserticola*. Phylogenetic distances between known PAL gene and assembled potential PAL sequences in *C*. *deserticola*.
(TIF)

S1 Dataset All primer sequences for 10 selected genes related to lignins biosynthesis pathway. (XLSX)

S2 Dataset All expressed transcripts with annotation in assembled *C*. *deserticola* stem transcriptome. (XLSX)

S3 Dataset GO annotation of all expressed transcripts in *C*. *deserticola* stem transcriptome. (XLSX)

S4 Dataset KEGG pathway annotation of all expressed transcripts with predicted ORF in *C*. *deserticola* stem transcriptome. (XLSX)

S5 Dataset Significantly changed KEGG pathways between *C*. *deserticola* and rice (tissues of callus and leaf). (XLSX)
