# Supplementary figures and images for: RNA-Seq Based De Novo Transcriptome Assembly and Gene Discovery of Cistanche deserticola Fleshy Stem
Source: PLoS One. 2015 May 4;10(5):e0125722. doi: 10.1371/journal.pone.0125722 (PMC4418726; doi:10.1371/journal.pone.0125722)

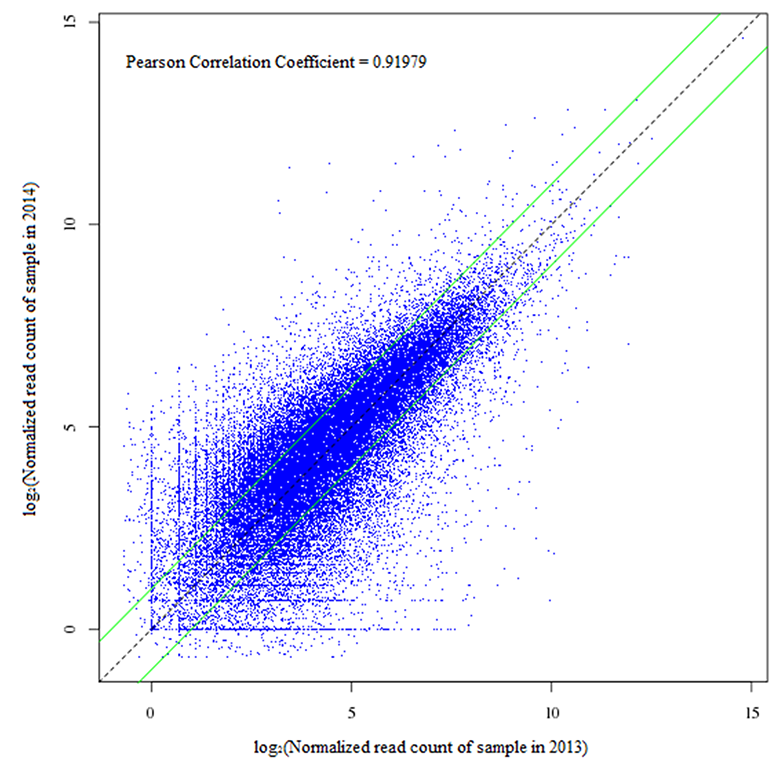

Supplement: S1 Fig — Normalized read count of two replicates (2013-year sample and 2014-year sample) were plotted, and high correlation showed reproducibility of our RNA-seq data. (TIF) [file pone.0125722.s001.tif]

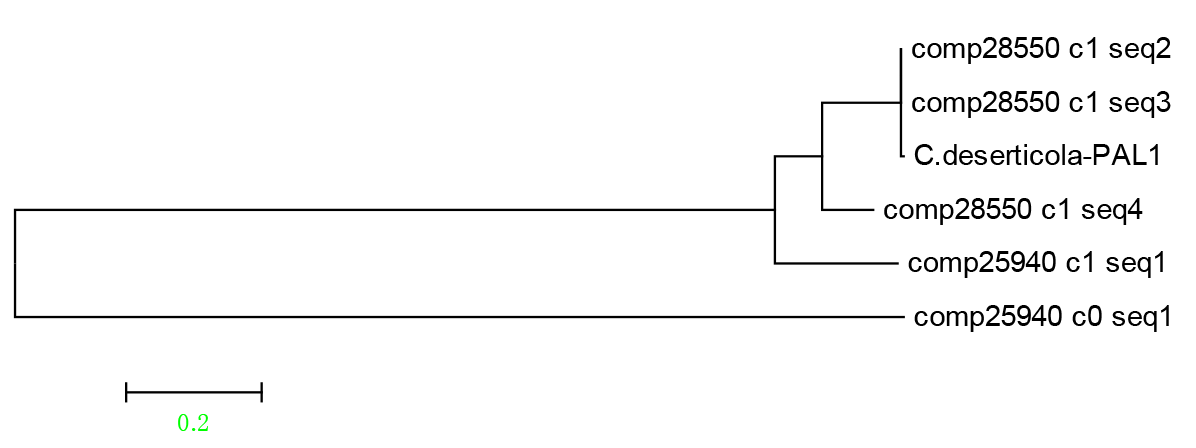

Supplement: S2 Fig — Phylogenetic distances between known PAL gene and assembled potential PAL sequences in C. deserticola. (TIF) [file pone.0125722.s002.tif]
